# Electrospun carbon nanofibre-assisted patterning of metal oxide nanostructures

**DOI:** 10.1038/s41378-022-00409-8

**Published:** 2022-06-29

**Authors:** Monsur Islam, Christian Dolle, Ahsana Sadaf, Peter G. Weidler, Bharat Sharma, Yolita M. Eggeler, Dario Mager, Jan G. Korvink

**Affiliations:** 1 Institute of Microstructure Technology, Karlsruhe Institute of Technology, Hermann-von-Helmholtz-Platz 1, 76344 Eggenstein-Leopoldshafen, Germany; 2 Microscopy of Nanoscale Structures & Mechanisms (MNM), Laboratory for Electron Microscopy (LEM), Karlsruhe Institute of Technology, Engesserstr. 7, D-76131 Karlsruhe, Germany; 3 Institut für Funktionelle Grenzflächen, Karlsruhe Institute of Technology, Hermann-von-Helmholtz-Platz 1, 76344 Eggenstein-Leopoldshafen, Germany

**Keywords:** Nanowires, Nanowires

## Abstract

This work establishes carbon nanofibre-mediated patterning of metal oxide nanostructures, through the combination of electrospinning and vapor-phase transport growth. Electrospinning of a suitable precursor with subsequent carbonization results in the patterning of catalyst gold nanoparticles embedded within carbon nanofibres. During vapor-phase transport growth, these nanofibres allow preferential growth of one-dimensional metal oxide nanostructures, which grow radially outward from the nanofibril axis, yielding a hairy caterpillar-like morphology. The synthesis of metal oxide caterpillars is demonstrated using zinc oxide, indium oxide, and tin oxide. Source and substrate temperatures play the most crucial role in determining the morphology of the metal oxide caterpillars, whereas the distribution of the nanofibres also has a significant impact on the overall morphology. Introducing the current methodology with near-field electrospinning further facilitates user-defined custom patterning of metal oxide caterpillar-like structures.

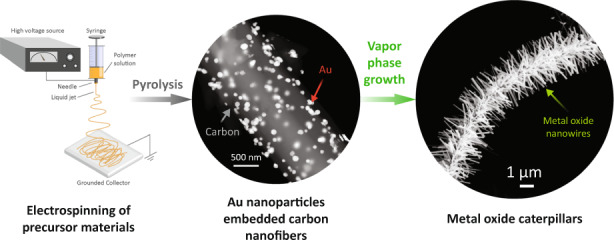

## Introduction

Nanostructured semiconductor metal oxides (NSMO) are an intensely investigated class of materials in the field of nanotechnology, due to their interesting morphologies, compositions, chemical, and physical properties. NSMOs are typically studied in the form of thin films. However, advances in nanotechnology have allowed the synthesis of zero-dimensional and one-dimensional metal oxide nanostructures, which have resulted in unprecedented performance improvement of the embedding electronic devices^1^. Particularly, studies of one-dimensional nanostructures of metal oxides have shown that, by engineering, one can tune the electronic structures, leading to a variety of changes in their chemical and physical properties. Several one-dimensional nanostructures, including nanowires, nanorods, nanotubes, nanofibres, nanobelts, nanocombs, and nanosprings, have been studied for several applications^2–6^. The engineering of one-dimensional metal oxide nanostructures can be achieved by customizing the nanoscale process of synthesis. Several synthesis methods have been developed to grow these nanostructures, which include vapor-phase transport (VPT), chemical vapor deposition, hydrothermal synthesis, microwave-assisted synthesis, and template-assisted synthesis^1,4,7^. Among these synthesis techniques, VPT has been widely used due to its low cost, simplicity, easy operation, and ability to grow a wide range of nanostructures.

The VPT process typically uses a catalyzed substrate, where the catalyst initiates and guides the growth of the nanostructures^8^. Furthermore, the epitaxial orientation between the one-dimensional nanostructure and the substrate yields a desired alignment of the nanostructures. Typically gold nanoparticles (AuNPs) are used as the catalysts in the VPT process, and the growth of the nanostructures occurs solely on the location of these catalysts^9^. Such characteristic of the VPT growth has triggered great interest in pattering metal oxide nanostructures in various geometries. For example, Greyson et al. demonstrated the fabrication of ordered arrays of zinc oxide (ZnO) nanowires by patterning of AuNP layer through photolithography, followed by VPT growth of the ZnO nanowires at the location of the patterned AuNPs^10^. He et al. reported patterning of vertically aligned ZnO nanowires by nanopatterning of a catalyst gold layer using atomic force microscopy nanomachining, followed by epitaxial growth of the ZnO nanowires in VPT method^11^. Other methods that have been employed to control the position of the catalyst nanoparticles for metal oxide nanostructure growth include deposition of Au through a shadow mask^12^, laser interference lithography^13^, and self-assembly nanosphere lithography^14^. However, all these methods are quite complex and require expensive infrastructure to pattern the catalyst layer. Furthermore, these methods only allow a two-dimensional positioning of the catalyst nanoparticles, which leads to two-dimensional arrangement of the metal oxide nanostructures after VPT growth. Here, we use electrospinning technology to achieve a three-dimensional distribution of catalyst nanoparticles and yield a three-dimensional arrangement of the metal oxide nanostructures in VPT growth mechanism.

Electrospinning is a method of drawing nanofibres from a polymer solution under a high electric field^15,16^. It allows the fabrication of fibers from a wide range of polymers with a high structural uniformity, a high surface area, and a high mechanical strength. The traditional electrospinning process results in the formation of a mat of randomly oriented fibers. However, the emergence of near-field electrospinning has made it possible to pattern in various engineered two-dimensional and three-dimensional geometries^17^. Furthermore, electrospinning allows to derive nanofibres of various other materials beyond polymers, which include carbon, metal oxides, ceramics, and composites. In fact, patterning of ZnO nanowires was also reported using electrospinning, where electrospinning facilitated the patterning of seed ZnO layer and subsequent hydrothermal growth of ZnO nanowires were achieved on the seed layer^18–20^. However, the VPT growth of metal oxide nanostructures using electrospun carbon nanofibres is of special interest here. Typically, electrospun carbon nanofibres are produced through electrospinning of a polymer precursor followed by a high-temperature carbonization step. Recent research works on polymer carbonization have demonstrated that three-dimensional arrangement of metal particles can be obtained while carbonizing a shaped metal precursor/polymer composite^21–23^. Therefore, the carbonization of electrospun fibers of a catalyst precursor/polymer composite can lead to a facile approach for patterning of the catalyst nanoparticles for the metal oxide growth in VPT process.

In this work, we report on the growth of metal oxide nanostructures in nanofibril arrangement by integrating the VPT growth route with the electrospinning process. We first pattern the catalyst AuNPs using electrospinning of the precursor materials, followed by carbonization. The resulted material was obtained as the substrate for the metal oxide growth in the subsequent VPT process. We demonstrate our patterning approach with ZnO nanostructures. We study the effect of the processing variables on the morphology of ZnO obtained after the VPT growth. We further demonstrate the growth of other metal oxides using our approach. We also exhibit the feasibility of user-controlled patterning of metal oxide nanostructures using the electrospinning-assisted approach.

## Results

**Fig. 1:**
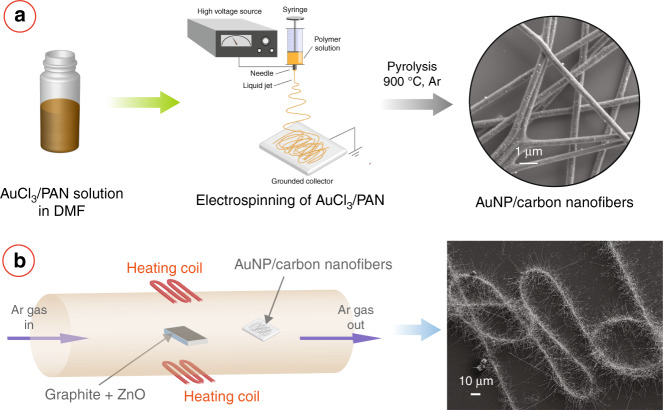
Fabrication scheme of electrospinning-assisted patterning of metal oxide nanostructures. Illustration of the fabrication process consisting of two main steps. **a** fabrication of AuNP/carbon nanofibres by electrospinning of AuCl_3_/PAN solution followed by carbonization; and **b** vapor-phase transport growth of metal oxide nanowires around the nanofibres yielding a caterpillar-like morphology.

Figure 1 illustrates the scheme of the patterning of metal oxide nanowires. Details of the fabrication process are provided in the Experimental section. Briefly, we first obtained AuNPs-decorated carbon nanofibres by using electrospinning of a gold chloride (AuCl_3_)/polyacrylonitrile (PAN) composite solution, followed by carbonization of the electrospun nanofibres at 900 °C for 1 h under a constant flow of argon gas. These AuNPs/carbon nanofibres were used as the catalyst template in the subsequent VPT growth, carried out in a tube furnace. In the VPT growth, a mixture of graphite powder and metal oxide powder was used as the source material, and the AuNPs/carbon nanofibres were used as the growth substrate. The VPT growth resulted in the growth of the metal oxide nanostructures on the nanofibril template. We chose ZnO as the metal oxide for our experiments due to its immense popularity in the scientific community. After the vapor-phase transport deposition, a white substance was obtained on the substrate at a distance of 12–22 cm from the reaction zone. Outside this region, no fibrous structure was observed.

### Effect of AuNPs in the growth of ZnO nanowires

**Fig. 2:**
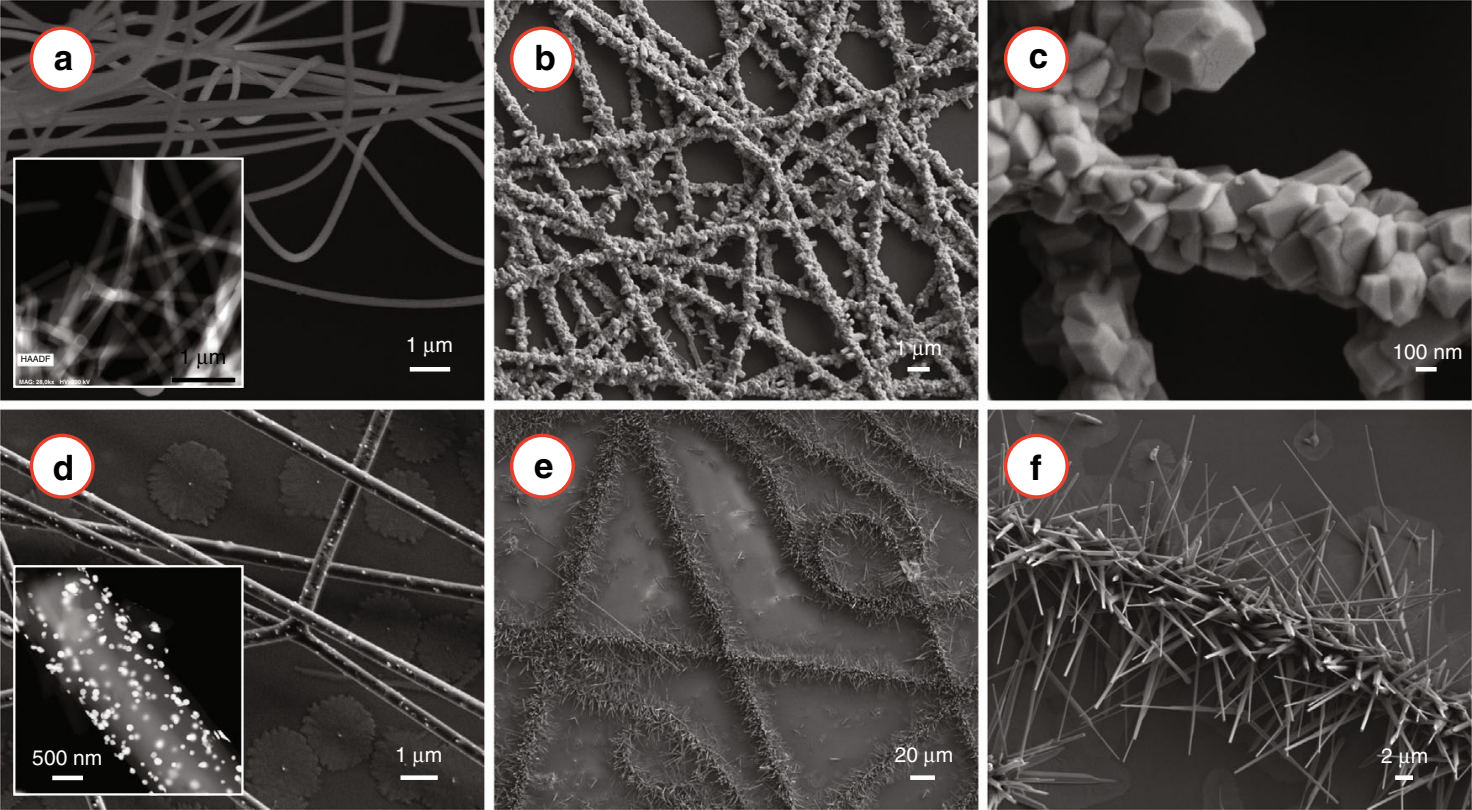
Role of AuNPs in the carbon nanofiber-assisted growth of ZnO nanostructures. **a** SEM of carbon nanofibres. Inset shows the HAADF STEM image of carbon nanofibres. **b**, **c** Low and high magnification SEM images of the ZnO nanostructures, respectively, obtained after vapor-phase transfer growth using the carbon nanofibres as the substrate. **c** SEM image of AuNPs/carbon nanofibres. Inset presents the HAADF STEM image of AuNPs/carbon nanofibres, showing the distribution of AuNPs within the carbon nanofibril matrix. The bright spots represent the AuNPs. **e**, **f** Low and high magnification SEM image of the caterpillar-like ZnO nanowires obtained using the AuNPs/carbon nanofibres.

Carbonization of the electrospun AuCl_3_/PAN nanofibres resulted in the formation of the AuNPs-decorated carbon nanofibres. X-ray diffraction (XRD) of the carbonized nanofibres (Fig. S1a) confirmed the formation of gold nanoparticles, as the XRD diffractogram featured sharp peaks of gold at 2*θ* = 38.1°, 44.4°, 64.5°, 77.6°, and 81.77°, which were matched with the International Centre for Diffraction Data (ICDD) powder diffraction file (PDF) number 04-0784. The presence of gold was further confirmed by the energy-dispersive X-ray spectroscopy (EDX) of the nanofibres (Fig. S1b). The distribution of the gold within the nanofibre was investigated using scanning transmission electron microscopy (STEM). Inset of Fig. 2d shows the high-angle annular dark-field (HAADF) STEM image of AuNPs/carbon nanofibres, depciting the distribution of the AuNPs within the nanofibril carbon matrix. The EDX mapping revealed that the proportion of carbon and gold present in the nanofibres was (C:Au=) 8.4. The average diameter of the electrospun fibers was 363.5 ± 41.8 nm. The presence of several nanoclusters of gold with a diameter ranging from a few nanometers to 100 nm was observed on the surface of the carbonized electrospun fibers during the electron microscopy analysis (Fig. S1c–f).

To investigate the effect of AuNPs, we performed the VPT growth experiments using electrospun carbon nanofibres with and without AuNPs. Figure 2a and its inset show the SEM and HAADF STEM image of electrospun carbon nanofibres, respectively. Figure 2b, c and e, f show the VPT growth results on the carbon and AuNPs/carbon nanofibres, respectively. Both carbon and AuNPs/carbon nanofibres facilitated the growth of ZnO, and the nanofibril pattern was retained after the growth, as shown in Fig. 2b, e. However, the morphology of the ZnO nanostructures was significantly different. The carbon nanofibres resulted in the growth of large crystals with irregular sizes (Fig. 2c). On the contrary, in the case of AuNPs/carbon nanofibres, one-dimensional ZnO nanostructures were grown radially outward from the center of the nanofibres, resulting in a caterpillar-like morphology, as shown in Figure 2f. Therefore, it can be inferred that it was the AuNPs present within the carbon matrix, which facilitated the nanowire growth in the nanofibril arrangement.

### Effect of source temperature on the growth of ZnO nanostructures

**Fig. 3:**
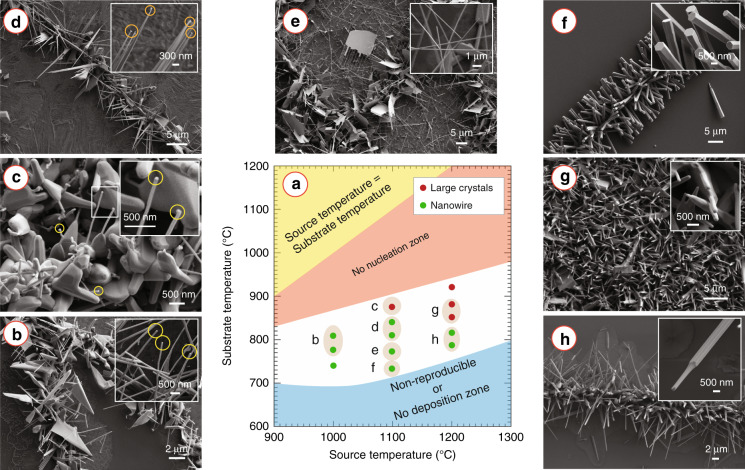
Effect of source and substrate temperature on the growth of ZnO nanostructures. **a** Shape diagram showing the effect of source temperature (*T*_H_) and substrate temperature (*T*_S_) on the morphology of the ZnO nanostructures. ZnO nanostructures grown at different growth conditions: **b** *T*_H_ = 1000 °C and *T*_S_ = 800 °C, **c** *T*_H_ = 1100 °C and *T*_S_ = 860 °C, **d** *T*_H_ = 1100 °C and *T*_S_ = 800–825 °C, **e** *T*_H_ = 1100 °C and *T*_S_ = 770 °C, **f** *T*_H_ = 1100 °C and *T*_S_ = 730 °C, **g** *T*_H_ = 1200 °C and *T*_S_ = 830–850 °C, and **h** *T*_H_ = 1200 °C and *T*_S_ = 770–800 °C. The substrate temperature was estimated from numerical simulation.

**Fig. 4:**
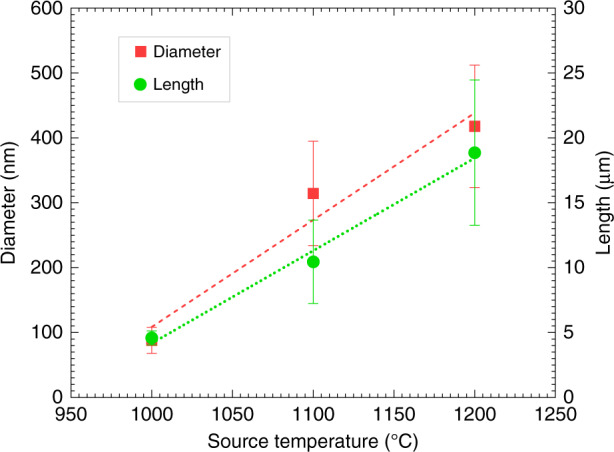
Dimensions of ZnO nanowires. Effect of source temperature on the dimensions of the ZnO nanowires.

Source temperature (which was equal to the temperature of the heating zone of the furnace (*T*_H_)) exhibited a significant influence on the morphology of the ZnO nanostructures. No caterpillar-like morphology was observed for a source temperature of 900 °C, as almost no nanowire growth around the nanofibril core was observed, as shown in Fig. SI2. A source temperature of 1000 °C yielded growth of ZnO nanowires (inset of Fig. 3b), featuring an average diameter of 88.0 ± 20.1 nm and a length of 4.59 ± 0.54 μm. These nanowires exhibited a significant side growth of ZnO at the base region, resulting in a nanosheet-like base region structure (Fig. 3b). The geometry of these nanosheet bases was irregular. As the source temperature increased to 1100 °C, the side growth of the nanowires reduced compared to a similar substrate temperature (*T*_S_), which was estimated from numerical simulation (Fig. SI3) and presented in Fig. 3a. The reduced side growth led to the formation of needle-like nanostructures of ZnO (Fig. 3d). For both the source temperatures, gold nanoparticle droplets were seen at the tip of many ZnO nanostructures (insets of Fig. 3b, d). However, no catalyst droplet was seen at the tip of the nanostructures obtained for the source temperature of 1200 °C (inset of Fig. 3h). At this source temperature, hierarchical nanorods were grown, where a smaller diameter faceted nanorod (diameter = 205 ± 74 nm) emerged from a larger diameter faceted nanorod (diameter = 418 ± 94 nm) as shown in Fig. 3h. These hierarchical nanorods featured a length of 19 ± 6 μm. Overall, the average diameter and length of the nanostructures increased linearly with the source temperature, which is plotted in Fig. 4.

### Material properties of ZnO

**Fig. 5:**
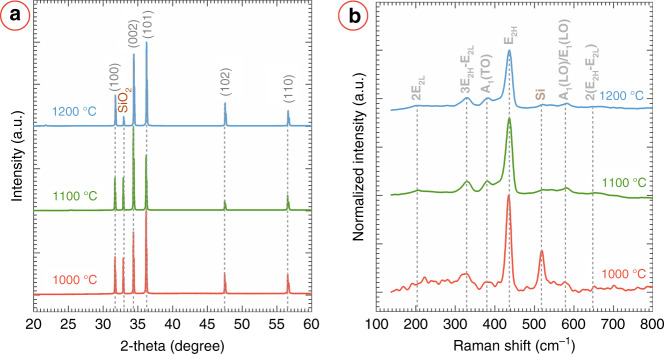
Material characterization of ZnO nanowires. **a** XRD diffractogram and **b** Raman spectrum of the ZnO nanostructures grown on the electrospun AuNP/carbon nanofibres using vapor-phase transport method.

Along with the ZnO morphology, the source temperature also exhibited a strong influence on the material properties of the grown ZnO nanostructures. We characterized the material properties using XRD and Raman spectroscopy. The results are presented in Fig. 5. In the XRD diffractogram, the peaks at 2*θ* = 31.84°, 34.44°, 36.23°, 47.52°, and 56.57° were indexed to the (100), (002), (101), (102), and (110) crystal planes of hexagonal ZnO, respectively, as shown in Fig. 5a. The peaks were matched to ICDD PDF number 36-1451. Along with the peaks of ZnO, a peak at *θ* = 32.91° was also observed, which was attributed to the SiO_2_/Si substrate^24^.

The sharp and high-intensity XRD peaks of ZnO suggested the formation of highly crystalline ZnO^25^. We calculated the lattice parameter *d*_002_ for the synthesized ZnO nanostructures at different source temperatures using Bragg’s equation. The *d*_002_ values are listed in Table 1, which are almost similar to the lattice parameter of unstressed bulk ZnO (*d*_002_ = 0.26 nm^26^). Furthermore, the dislocation density (*δ*) and microstrain (*ϵ*) calculated from the XRD diffractogram featured a low value (Table 1) and did not vary significantly with the source temperature. The low value of *δ* and *ϵ* confirmed very few lattice defects and good crystalline quality of the ZnO nanostructures grown in our work. We further calculated the texture coefficients (TC_*hkl*_) of different planes to understand the phase contribution within our ZnO nanostructures. The calculated texture coefficients in the (100), (002), and (101) planes are presented in Table 1. For all the source temperatures, the (002) plane featured the highest texture coefficients, with a maximum value of 2.42 for the source temperature of 1100 °C. This suggested a strong preferential growth along the *c*-axis, which led to the nanowire, nanoneedle, and nanorod morphologies of the ZnO^27^. However, the texture coefficients in the (100) and (101) planes indicated a significant side growth of the ZnO nanostructures. However, the side growth seemed to decrease with the increase in source temperature, as the texture coefficient value in the (100) exhibited a decreasing trend with the source temperature.

**Table 1:** Crystallographic properties of the ZnO nanostructures depending on the source temperature.

Source temperature (°C)	d_002_ (nm)	*δ* × 10^−4^ (nm^−2^)	*ϵ* × 10^−3^	TC_100_	TC_002_	TC_101_
1000	0.26	30	2.04	0.74	1.57	0.93
1100	0.26	31	2.09	0.72	2.42	0.70
1200	0.26	29	2.02	0.59	1.82	0.93

According to group theory, the optical phonons at the Γ point of the Brillouin zone are described as shown in Equation (1) ^28,29^.

1 $${{{\Gamma }}}_{{{{\rm{opt}}}}}=1{A}_{1}+2{B}_{1}+1{E}_{1}+2{E}_{2}$$

For a single crystal ZnO, *A*_*1*_ and *E*_*1*_ modes are polar, Raman and infrared active, and split into transverse optical (TO) and longitudinal optical (LO) branches^30,31^*. E*_*2*_ mode is non-polar and only Raman-active, and consists of low- and high-frequency phonon modes (*E*_*2L*_ and *E*_*2H*_ respectively). The *B*_*1*_ mode is silent to infrared and Raman. Hence, no *B*_*1*_ peak is expected in the Raman spectra of the synthesized ZnO. The most dominant Raman peak for the ZnO nanostructures synthesized here was located at ~436 cm^−1^ (Fig. 5b), which was assigned to *E*_*2H*_ phonon-mode. A red-shift and a broadening of the *E*_2*H*_ peaks were observed with the increasing source temperature (see Table S1), although the variation between the samples for 1100 °C and 1200 °C was minimal. Such variation of the *E*_2*H*_ peak may attribute to the surface disorders of the nanostructures^32^. . However, the strong *E*_*2H*_ peak confirmed the high crystalline quality of the synthesized ZnO, which is in agreement with the XRD diffractogram. The other significant Raman peaks were present at 100 cm^−1^, 383 cm^−1^, and 582 cm^−1^, which were indexed to low-*E*_*2*_ (*E*_*2L*_), *A*_*1*_(TO), and *A*_*1*_(LO)/*E*_*1*_(LO), respectively. The *E*_*1*_(LO) peak is associated with the defects in the samples^33^. The intensity ratio of *E*_*1*_(LO) and *E*_*2H*_ also exhibited an increasing trend with the source temperature (Table S1), which further suggested increasing surface defects with source temperature^34^. The second order phonon-mode 2*E*_*2L*_ emerged at 206 cm^−1^. The peaks at 330 cm^−1^ and 660 cm^−1^ were attributed to the multi-phonon scattering modes 3*E*_*2H*_-*E*_*2L*_, and 2(*E*_*2H*_-*E*_*2L*_), respectively. All the Raman peaks demonstrated excellent agreement with previously published reports, which further confirmed the wurtzite-type structure of the synthesized ZnO^30,31,35,36^.

**Fig. 6:**
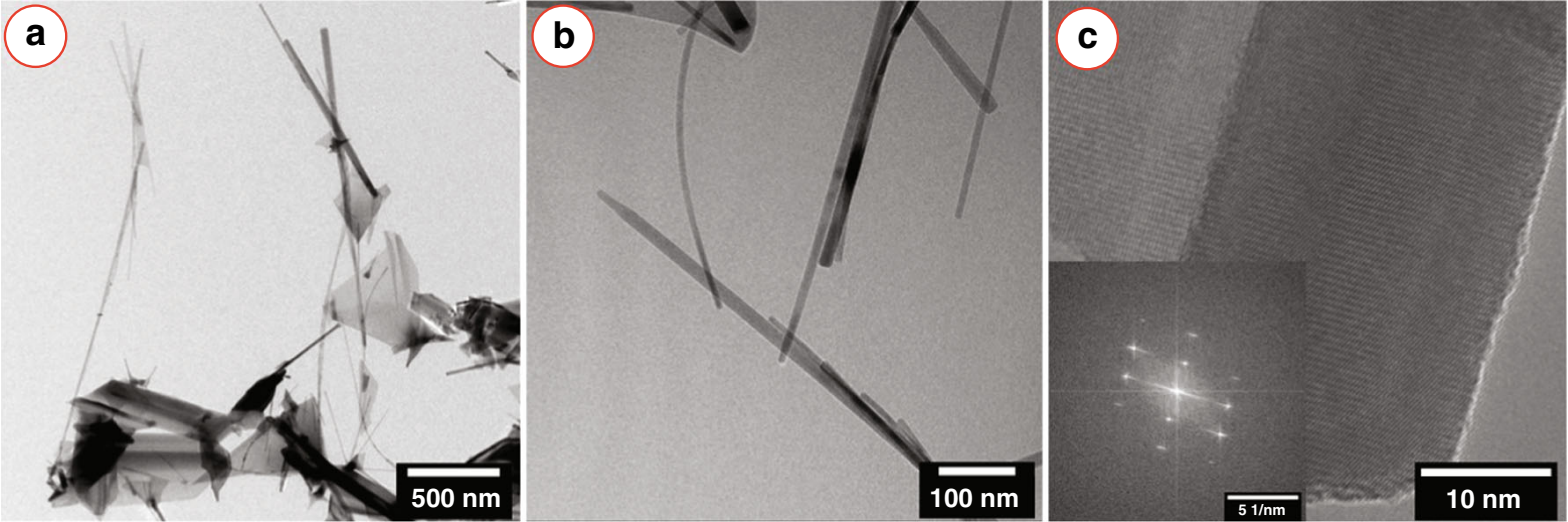
TEM of ZnO nanowires. **a** Bright field TEM overview image showing ZnO nanowires with several micrometer length and layered crystalline ZnO nanosheets. **b** Close up of some individual nanowire structures. **c** High-resolution TEM of individual ZnO nanowire with pronounced crystallinity, as evident from the fast Fourier transform (inset). The particular sample for TEM analysis was prepared using a source temperature of 1000 °C

We further performed transmission electron microscopy (TEM) of the ZnO caterpillars. Figure 6 shows fragments of the ZnO nanostructures grown in our study, the pristine ZnO nanostructures might have fragmented during the sample preparation for TEM. Nevertheless, both nanowires and broad nanosheet morphologies were observed with TEM, as presented in Fig. 6a. The nanosheet morphologies were attributed to the base region of the nanowires, as mentioned in the earlier sections. The nanowire fragments studied using TEM featured lengths exceeding several micrometers and diameters below 100 nm (Fig. 6a, b). No Au catalyst particles were apparent on the investigated wires, which might be a consequence of the fragmentation of the pristine sample. The ZnO nanowires exhibited a pronounced crystallinity, as evidenced by the high-resolution TEM image. The corresponding fast Fourier transform pattern (Fig. 6c) further evidences the ordered single crystalline structure of the obtained ZnO nanowires based on the observed HRTEM image.

### Effect of substrate position on the morphology of ZnO nanostructures

The morphology of the ZnO nanostructures grown in the nanofibril arrangement varied with the distance from the heating zone. Figure 3c–f shows the change of morphology of the ZnO nanostructures, as the distance from the heating zone increased, and the source temperature was 1100 °C. Within a distance of 12–13 cm from the source, the ZnO nanostructures featured a broad and faceted nanorod (NR)-shapes, which featured an average diameter of 269.3 ± 47.6 nm and an average length of 2.09 ± 0.33 μm. From many of these 3D faceted structures, 1D nanowires (NWs) emerged, which had an average diameter of 61.7 ± 9.0 nm and an average length of 1.54 ± 0.31 μm. At the tip of the nanowires, Au catalyst droplets were observed, which are indicated by the circles in Fig. 3c. Such catalyst droplets were also observed in the nanostructures grown in the region of 13–14 cm from the source (inset of Fig. 3d). The nanostructures grown in this region resembled needle-like nanowires, featuring a broad and faceted base (average diameter = 314.4 ± 80.4 nm) and a sharp tip. The average length of these nanowires was 10.46 ± 3.21 μm. Beyond this region, the ZnO nanostructures did not feature any catalyst droplet at their tips. A significant side growth of ZnO occurred for many of these nanowires near their base region, resulting in a wide nanosheet-like base region. The width of these nanosheet bases increased as the distance from the source increased. In many cases, the proximity of the nanowires led to the side growth of ZnO merging the base regions of the nanowires, forming a comb-like morphology (Fig. 3e). Furthermore, as the distance increased, many faceted nanorods featuring a hexagonal cross-section started to emerge. Growth of only ZnO rod-like shapes with a diameter of 898.9 ± 216.4 nm and length of 9.75 ± 1.56 μm was observed within a distance of 20–21 cm from the source (Fig. 3f). Beyond this range, either no growth or the growth of non-reproducible irregular structures was observed.

### Feasibility of patterning other metal oxides

**Fig. 7:**
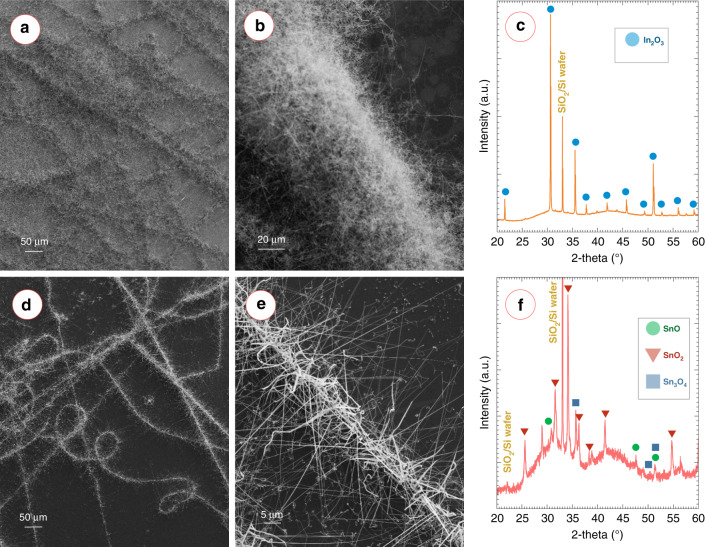
Patterning of different metal oxide caterpillars. **a** Low magnification and **b** high magnification SEM image of indium oxide nanostructures obtained after AuNP/carbon nanofibre-assisted VPT growth, showing the caterpillar-like morphology. **c** XRD diffractogram of indium oxide caterpillars showing sharp peaks of cubic In_2_O_3_. Peaks of SiO_2_/Si were also observed. **d** Low magnification and **e** high magnification SEM image of tin oxide caterpillars. **f** XRD diffractogram of tin oxide caterpillars showing the presence of tetragonal SnO and SnO_2_, and triclinic Sn_3_O_4_ phases.

To demonstrate the versatility of our approach, we also attempted to pattern other metal oxides, including indium oxide (In_2_O_3_) and tin oxide (SnO_2_). We used a source temperature of 1100 °C for these experiments. Similar to the results of ZnO, the nanofibril arrangement was retained for both In_2_O_3_ and SnO_2_, as shown in Fig. 7a and d, respectively. Long nanowires of In_2_O_3_ and SnO_2_ emerged radially from the nanofibril axes (Fig. 7b and e, respectively), resulting in the caterpillar-like morphology as obtained in the case of ZnO. Figure 7c and f present the XRD diffractograms of the VPT-growth materials, which confirmed the formation of cubic phase In_2_O_3_ (ICDD PDF no. 71-2194)^37^ and tetragonal SnO_2_ ((JCPDS card no. 72-1147)^38^ nanostructures, respectively. Presence of tetragonal SnO (ICDD PDF no. 078-1913) and triclinic Sn_3_O_4_ ((ICDD PDF no. 16-0737) were also indicated along with SnO_2_ for the tin oxide nanowires. It should be noted that there were deviations of the Sn_3_O_4_ peaks from the above-mentioned PDF number, which were within the errors as expressed by Lawson^39^.

### 2D patterning of nanowires

**Fig. 8:**
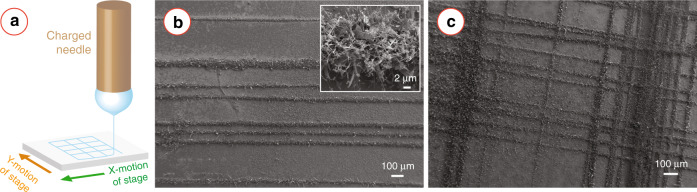
Near-field electrospinning-assisted patterning of metal oxide nanowires. **a** Schematic of near-field electrospinning. Examples of near-field electrospinning-assisted patterning of ZnO nanostructures in form of **b** parallel lines and **c** rectangular grids. Inset of **b** shows the high magnification image of the ZnO nanostructures within the parallel lines.

So far, we have introduced the use of randomly oriented nanofibres to synthesize metal oxide caterpillars. To achieve an ordered patterning, we used near-field electrospinning to pattern the AnNPs/carbon nanofibres in parallel straight lines or rectangular grids. VPT growth of metal oxides using these ordered patterns of AnNPs/carbon nanofibres led to ordered patterning of the metal oxide caterpillars. Figure 8b shows the parallel straight lines of ZnO caterpillar, whereas the rectangular grid patterning is shown in Fig. 8c. The ZnO caterpillars featured both long nanowires and nanosheet morphologies as shown in the inset of Fig. 8b.

## Discussion

Carbonization of the electrospun AuCl_3_/PAN nanofibres resulted in the formation of AuNPs-decorated carbon nanofibres. During the heat treatment, PAN decomposed to a carbon-rich material at a temperature higher than 350 °C^40,41^. With further increase in the temperature, heteroatoms such as oxygen, hydrogen, and nitrogen escaped from the matrix, and condensation and recrystallization of the carbon occurred, resulting in a glass-like carbon material^42,43^. During this carbonization process, the AuCl_3_ reduced to form AuNPs within the carbon matrix^44^, which acted as the catalyst nanoparticles in the subsequent vapor-phase transport growth of the ZnO nanostructures. During the carbonization of a polymer-metal precursor composite, metal nanoparticles tend to migrate to the carbon surface and coalesce with adjacent metal nanoparticles to form clusters or larger particles^21–23^. This phenomenon also occurred in our work, as several nanoclusters of gold were observed on the surface of the carbon nanofibres, as indicated in Fig. S1c–f. The gold nanoparticles within the carbon nanofibres further led to a preferential growth of the metal oxide nanostructures radially outward from the nanofibril axis, resulting in the caterpillar-like structures.

The growth of the metal oxide (MO_*x*_) nanostructures started with the formation of metal vapor through carbothermal reduction (Eq. 2) of the MO_*x*_ powder by graphite particles in the heated zone of the furnace:

2 $${{{{\rm{MO}}}}}_{x}({{{\rm{s}}}})+{{{\rm{xC(s)}}}}\to {{{\rm{M(g)}}}}+{{{\rm{xCO(g)}}}}$$

The argon gas carried the metal vapor to the deposition zone, where the metal vapor reacted with the residual oxygen atoms in the furnace tube to facilitate the metal oxide nanostructure growth. In a VPT process, the growth of MO_*x*_ nanostructures has two important aspects: nucleation and growth. Nucleation occurs through agglomeration of metal ions and oxygen ions due to minimization of surface energy. A nucleation site with the minimum size is possible when the participating ions overcomes the maximum energy barrier ${{\Delta }}{G}_{N}^{* }$, which relates to the surface energy per unit area (*γ*) and the Gibbs free energy per volume (Δ*G*^0^) (Eq. 3)^45,46^. Based on this, Choopun et al. derived a relation for the nucleation probability (*P*), which relates to ${{\Delta }}{G}_{N}^{* }$, nucleation temperature (T) and Boltzmann constant (*K*_B_)^45^, as shown in Eq. (4).

3 $${{\Delta }}{G}_{N}^{* }=\frac{16\pi {\gamma }^{3}}{3{({{\Delta }}{G}^{0})}^{2}}$$

4 $$P={\rm{exp}}(-\frac{{{\Delta }}{G}_{N}^{* }}{{k}_{\rm{B}}T})$$

There are three possible schemes in VPT for the growth of MO_*x*_ nanostructures: (i) catalyst-free nucleation and growth, (ii) catalytic nucleation and catalyst-free growth, and (iii) catalytic nucleation and growth^47^. In scheme (i), the metal reacts with oxygen in vapor phase to form the metal oxide and condensates on a surface to form MO_*x*_ islands. These MO_*x*_ islands act as the nucleation sites for the subsequent growth of MO_*x*_ in a vapor-solid mechanism^48,49^. This phenomenon was observed, when we used carbon nanofibres without any catalyst gold nanoparticles. Carbon nanofibres feature lower surface energy compared to SiO_2_/Si substrate. According to Eqs. (3) and (4), lower surface energy facilitates the nucleation of metal oxides. Therefore, preferential nucleation of ZnO occurred at the sites of the carbon nanofibres. However, no epitaxial growth of the ZnO was observed due to the absence of guiding catalyst nanoparticles. In contrast, rapid condensation of ZnO occurred at the nucleation sites, leading to the formation of nanofibres of multi-faceted large crystallites, as shown in Fig. 2b, c. Catalyst nanoparticles play a crucial role in schemes (ii) and (iii). In scheme (ii), nucleation of metal oxide is influenced by the Au catalyst nanoparticles. Au atom has a strong binding affinity to metal oxides. For example, the binding energy of Au on the polar surface of ZnO is 0.94 eV^50^. Due to the low interfacial surface energy, the metal vapor atoms impinge and stick to the catalyst Au nanoparticles^51,52^. The metal atoms further react to the residual oxygen atoms to form MO_*x*_ nuclei, which further provide preferential adsorption sites for the upcoming metal and oxygen gaseous species yielding growth of MO_*x*_ nanostructures in vapor-solid mechanism^53,54^. The scheme (iii) is governed by the vapor-liquid-solid mechanism, where the process starts with the formation of a liquid alloy through a reaction between the metal vapor atoms and catalyst AuNPs^55,56^. This alloy allows preferential accommodation of the gas phase precursors. Supersaturation of the metal at the alloy droplet leads to strong precipitation of the metal atoms at the interface of the liquid droplet and the substrate, which further allows local oxidation and epitaxial growth of MO_*x*_ nanostructures^49,57^. The alloy droplet remains at the tip, guiding the growth of the MO_*x*_ nanostructure. In this present work, the MO_*x*_ nanostructures obtained using the AuNP/carbon nanofibres grew in the schemes (ii) and (iii). Both AuNPs and carbon facilitated the preferential nucleation of the MO_*x*_, whereas the AuNPs led to the unidirectional growth of the MO_*x*_ nanostructures in either VLS or VS manner. Furthermore, the uniform distribution of the AuNPs within the nanofibril templates resulted in radially outward growth in all directions from the nanofibril axes, yielding the caterpillar-like morphology.

The morphology of individual nanostructures with the MO_*x*_ caterpillar varied depending on the experimental conditions. The source temperature and the distance of the substrate from the source were the two most dominating factors in determining the morphology of the MO_*x*_ nanostructures, as evidenced in this work, and are in well agreement with previous reports^9,47,54,58,59^. Figure 3a presents a shape diagram (inspired from the work by Wongchoosuk et al.^47^), which depicts the role of source temperature and substrate temperature on the evolution of different morphologies of ZnO. Source temperature determines the flux density of the metal vapor generated by the carbothermal reaction between the MO_*x*_ and graphite powder. A minimum concentration of the vapor flux is required to yield the nucleation of MO_*x*_ nanostructures. The flux density of the metal vapor increases with the source temperature^47,60^. Therefore, higher source temperature provides an excess amount of metal atoms to the growth area for a given growth temperature, which results in a higher supersaturation of the metal atoms at the catalyst-alloy liquid interface^54^. This phenomenon was also observed in our experiments with the ZnO caterpillars. We observed that no nanowire growth occurred below a source temperature 1000 °C. Furthermore, the diameter and the length of the ZnO nanostructures increased with the source temperature as shown in Fig. 4, which was a direct consequence of the increased flux density. Additionally, the increased flux density resulted in the transformation of the ZnO morphology from nanowires to nanorods for the temperature increment from 1000 °C to 1200 °C (Fig. 3).

The substrate temperature relates to the source temperature and the distance of the substrate from the source. In our experiments, the substrate was placed downstream of the heated zone of the furnace. The downstream temperature decreases with the distance from the heating zone. Figure S3 illustrates this temperature distribution in the furnace tube, as obtained from the numerical simulation. The numerical simulation further estimated that the temperature decreased with a rate of ~15 °C cm^−1^ on the substrate (Fig. S3c). This drastic temperature change on the substrate led to the morphological changes of the ZnO nanostructures for a given source temperature, as shown in Fig. 3. In this work, for a substrate temperature ranging from 800 °C to 850 °C, the growth of the ZnO nanostructures was mainly governed by the gold-assisted vapor-liquid-solid mechanism, the Au–Zn alloy nanoparticle were observed at the tip of the ZnO nanostructures, as shown in Fig. 3b–d. As the substrate temperature decreases, the condensation rate of the growth species increases, promoting the growth of the ZnO nanostructures more in a vapor-solid mechanism^9,47^. In our samples, for a substrate temperature below 800 °C, the vapor-solid mechanism gradually became more dominant over the vapor-liquid-solid mechanism. Supporting this, no alloy nanoparticle was observed at the tip of the ZnO nanostructures (Fig. 3e, f). For both the vapor–liquid–solid and vapor–solid mechanisms, the growth direction of the ZnO nanostructures was predominantly along the [0001] direction (perpendicular to the hexagonal facet). This was expected because the sticking coefficient of the Zn atoms is highest to the hexagonal facet compared to the other facets^61^. Although a unidirectional growth of the ZnO along the [0001] direction leading to nanowires was expected, growth in the non-[0001] directions was also observed in almost all the ZnO nanostructures obtained here, leading to a needle or spear-like morphology. However, the growth in the non-[0001] directions was mainly observed towards the bases of the ZnO nanostructures, and the growth rate in these directions was significantly lower than that of [0001] direction. Cheng et al. proposed that high flux density of the Zn atoms were the reasons behind the growth of the side facets^54^. They mentioned that at a high Zn vapor flux density, excess Zn atoms could attach to the side faces of the already formed ZnO nuclei, promoting secondary nucleation and growth of the ZnO nanostructures. Kumar et al. also reported that similar secondary nucleation and growth were higher at the bases due to the base region being the longest in the entire growth period^9^. We further hypothesize that the high density of the AuNPs catalysts in the nanofibre templates might have resulted in a higher flux density of the Zn atoms at the nanofibril surface, leading to higher secondary growth of the ZnO nanostructures towards the base regions. The rate of the secondary growth on the non-[0001] facets increases substantially with the increase in the substrate temperature^9^. For a substrate temperature greater than 825 °C, coalescence of the ZnO nanostructures bases was observed due to high secondary growth of the nanostructures, which led to the formation of the non-uniform and large crystallite structures. Examples of these occurrences are shown in Fig. 3c, g, and identified by red circular symbols in the shape diagram in Fig. 3a.

For a substrate temperature lower than 700 °C, no ZnO nanowire growth was observed, despite retaining the fibril pattern. Rather, non-uniform and non-reproducible ZnO structures were observed on the substrate. This lower substrate temperature was achieved at a relatively distant location from the source. At these farther distances, the vapor flux density of the Zn atoms is expected to be significantly lower^62^. A lower substrate temperature and a lower Zn flux density did not provide suitable conditions for the preferential accommodation of Zn atoms on the gold catalyst nanoparticles, as in the case of vapor-liquid-solid and vapor-solid growth. Therefore, non-catalytic nucleation and growth might have occurred at random places via direct condensation of the Zn vapor and subsequent oxidation. Similar growth of the ZnO was also observed at the inner wall of the tube (inset of Fig. S3a). This self-catalytically grown ZnO featured a mixture of morphologies, including nanowires, nanosheets, and nanoneedles, as shown in (Fig. S4a, b).

As we mentioned before, the final morphology of ZnO nanostructure was the combined effect of source temperature and substrate temperature. However, the final morphology of the ZnO nanostructures seemed to be more sensitive to the position on the substrate, which was attributed to the temperature gradient on the substrate and was in agreement to previous publications^62,63^. Similar phenomena were also observed for In_2_O_3_ and SnO_2_. However, variation of the morphology depending on source temperature and substrate temperature was different for different metal oxides. Here, we only demonstrated the feasibility of patterning of nanostructures of other metal oxides using In_2_O_3_ and SnO_2_. A detailed individual study similar to ZnO is needed for each metal oxide to fully elucidate the morphological variations.

Other processing variables, including carrier gas, carrier gas flow rate, source material, substrate properties, and inner diameter of the tube furnace, only have a minor influence on the final morphology of MO_*x*_ nanostructure^54,64–68^. In our work, we further noticed that the distribution of the AuNPs/carbon nanofibres had a significant effect on the morphology of the MO_*x*_ nanostructures. Along with the metal vapor flux and the substrate temperature, a denser distribution of the nanofibres was also observed to promote secondary growth of the MO_*x*_ nanostructures. The close proximity of the nanofibres yielded coalescence of the base regions of the MO_*x*_ nanostructures from the neighboring fibrils, often causing the disappearance of the nanofibril patterns. A few examples are shown in Figs. 3c, g and S5. Furthermore, a dense forest of MO_*x*_ nanostructures consisting of various morphologies including nanowires, nanorods, and nanosheets (Fig. S5) was observed stemming out of the coalesced MO_*x*_. We hypothesize that the coalesced base region might have created new nucleation sites, from which the growth of the 1D nanostructures of MO_*x*_ occurred in a self-catalytic mechanism. The evolution of these new nucleation sites at these coalesced forms often led to the synthesis of novel morphologies. Figure S5c shows an example, where the arrangement of larger crystals of ZnO at the tip of a nanowire caused ZnO nanoflower-like morphology. However, these novel morphologies were often non-reproducible.

Even though the starting material in the vapor-phase transport growth was AuNPs/carbon nanofibres, no carbon was detected in the final nanostructures obtained after VPT growth. The carbon within the template nanofibres might have oxidized to form CO/CO_2_ during the growth of the MO_*x*_ nanostructures due to the high oxygen content in the process. The oxidation of the carbon might have created a localized high concentration of CO/CO_2_, which might further fasten the oxidation rate of the incoming metal atoms. This might have further promoted the secondary nucleation and growth of the MO_*x*_ nanostructures, resulting in a thick base region for the MO_*x*_ nanowires. In the absence of AuNPs, this secondary growth yielded the formation of large crystals, as shown in Fig. 2b,c. Nevertheless, the main contribution of the AuNPs/carbon nanofibres was to supply the catalyst gold nanoparticles within the nanofibril frame, which ultimately facilitated the growth of the MO_*x*_ nanostructures. It should be mentioned here that the carbonization of the precursor AuCl_3_/PAN nanofibres allows for a simpler method to achieve the catalyst arrangements. The current state-of-the-art methods use already synthesized AuNPs for the growth of MO_*x*_ nanostructures in VLS/VS mechanism. However, the synthesis of AuNPs is a complex process, which involves complicated, carefully monitored, and time-consuming reduction chemistry^69–72^. In comparison, the one-step carbonization process allowed for the synthesis of the AuNPs through carbothermal reduction of the gold salt in a facile manner.

The traditional electrospinning results in a random distribution of nanofibres. Conversely, near-field electrospinning allows user-controlled patterning of two-dimensional geometries due to its high precision in nanofibre deposition^17,73^. In this work, we also demonstrated the feasibility of user-controlled patterning of the metal oxide caterpillars using near-field electrospinning, as shown in Fig. 8. This is, to the best of our knowledge, the first report of user-controlled patterning of metal oxide nanostructures. Here, we used a rotating drum as the moving platform, which limited our patterning to simple geometries with parallel lines. However, replacing the rotating drum collector with a high-speed X–Y stage can allow for complex two-dimensional geometries^17^, which can further lead to the complex 2D patterning of metal oxide caterpillars. This can be a facile and inexpensive alternative to the state-of-the-art lithography-assisted patterning of metal oxide nanostructures. The user-controlled patterning of metal oxide caterpillars can lead to the fabrication of functional devices with superior performances in various applications, including sensors, photodetectors, and solar cells. Furthermore, the high precision of near-field electrospinning can also lead to the fabrication of single caterpillar-based devices. Single nanofibre-based devices have been reported to perform better in comparison to nanofibril mesh^74,75^. A single MO_*x*_ caterpillar is expected to perform even better in comparison to a single MO_*x*_ nanofibre, due to the caterpillar morphology of the MO_*x*_. However, an extensive study is needed to fully elucidate the performance of the MO_*x*_ caterpillars to validate our speculation.

## Conclusion

This work demonstrated the patterning of metal oxide nanostructures by integrating electrospinning and vapor-phase transport growth. To facilitate the patterning, we first patterned the catalyst gold nanoparticles by electrospinning of a gold precursor/PAN solution, followed by carbonization. The resulting gold nanoparticle-decorated carbon fibers were used as the substrate material for the growth of metal oxide nanostructures in the subsequent VPT process. The presence of gold nanoparticles within the carbon fibers resulted in the growth of one-dimensional metal oxide nanostructures radially outward from the nanofibril axis, yielding a caterpillar-like morphology. We demonstrated the synthesis of the caterpillar structures using ZnO, In_2_O_3_, and SnO_2_. The source temperature and the substrate temperature played the most crucial roles on defining the morphology of the metal oxide caterpillar. However, the distribution of the nanofibres also had a significant impact on the overall morphology. We further demonstrated the feasibility of user-controlled patterning of the metal oxide caterpillars using near-field electrospinning. The user-controlled patterning of metal oxide caterpillars has high potential toward achieving the fabrication of functional devices containing crystallites with ultra-high surface-to-volume ratio.

## Experimental

### Materials

We used polyacrylonitrile (PAN) as the precursor for the carbon nanofibres. Gold chloride (AuCl_3_) was used as the source for the gold nanoparticles, which were used as the catalyst for the growth of the ZnO nanostructures. PAN (molecular weight: 150,000; Catalog number: 181315), graphite powder (Catalog number: 282863) and ZnO powder (Catalog number: 544906) were purchased from Sigma Aldrich, Germany. AuCl_3_ (Catalog number: ACRO318645000) and N,N Dimethylformamide (DMF; Catalog number: 1.03053.2511) were purchased from VWR, Germany. SiO_2_/Si wafers were used as the substrate for electrospinning.

### Electrospinning of the precursor solution

To prepare an electrospinnable precursor solution for AuNP/carbon nanofibres, we first mixed PAN and AuCl_3_ powder in a PAN to AuCl_3_ ratio of 10:1. The powder mixture is dissolved in DMF by stirring overnight on a magnetic stirrer at 500 rpm and 50 °C to yield a yellowish colored 10% (wt%) AuCl_3_/PAN solution as the spinning solution. We electrospun the AuCl_3_/PAN solution using a custom-built electrospinning set-up to obtain a randomly distributed nanofibre matrix. The parameters used for the electrospinning process included a voltage of 10 kV, a needle to collector distance of 10 cm and a flow rate of 10 μL min^−1^. The electrospun fibers were collected on a piece of SiO_2_/Si substrate. For patterning of the ZnO nanostrucures, we used a near-field electrospinning-based direct writing system. Pieces of SiO_2_/Si substrate with an area of 1 cm × 1 cm attached to a rotating drum (diameter = 15 cm) were used here as the movable substrate for patterning of the nanofibres. Near-field electrospinning of the solutions were achieved using a voltage of 1 kV, a distance from the needle tip to the rotating substrate of 1 cm, a drum speed of 120 rpm and a flow rate of 10 μL min^−1^. The obtained AuCl_3_/PAN nanofibres were stabilized on a hot plate at 95 °C for 4 h followed by heating for another 1 h at 200 °C. The fibers turned to dark brown color after stabilization.

### Carbonization of AuCl_3_/PAN nanofibres

The stabilized electrospun AuCl_3_/PAN nanofibres were carbonized in a horizontal tube furnace (Carbolite Giro) to obtain the template AuNP/carbon nanofibres. The carbonization recipe used in our work was adapted from a process popularly used in carbon microelectromechanical systems (C-MEMS) technology^76–80^. Briefly, the process involved three steps: heating from room temperature to 900 °C with a heating rate of 5 °C min^−1^, dwelling at 900 °C for 1 h, and cooling down to room temperature with natural cooling. A constant flow of Argon gas (flow rate = 0.8 L min^−1^ was maintained throughout the entire process.

### Growth of ZnO nanostructures

To facilitate the gas phase transport growth of the metal oxide nanostructures on the AnNP/carbon nanofibres, we first mixed the ZnO nanopowders with the graphite powder with a ZnO:graphite weight ratio of 6:5. The powder mixture was taken in an alumina crucible and kept it at the central heating zone of the tube furnace. The template nanofibres on the SiO_2_/Si substrate were kept at a distance from the source powder mixture in the downstream of the furnace. The temperature of the heated region of the furnace (T_H_ = source temperature) was increased to a final temperature ranging from 900 °C to 1200 °C with a heating rate of 5 °C min^−1^ under a constant argon gas flow with a flow rate of 0.3 L min^−1^. A dwell time of 30 min at the final temperature was used for the growth of the metal oxide nanostructures.

### Characterization

Electrospun carbon and AuNPs/carbon nanofibers were characterized for material composition and nanoparticle distribution using EDX mapping and STEM imaging on a FEI Osiris microscope operated at 200 kV, collecting data with a SuperX detector and employing Bruker’s Esprit software. To visualize the elemental distribution the integrated net counts are plotted on a color scale without any further processing. The morphology of the deposited ZnO nanostructures was characterized using scanning electron microscopy (SEM; Carl Zeiss AG–SUPRA 60VP). To further characterize the morphological and structural properties of the nanostructures, TEM was carried out on a TFS CM200 FEG/ST transmission electron microscope operated at 200 kV, equipped with a TVIPS F416 4k CMOS camera. The material properties of the deposited ZnO were characterized using Raman spectroscopy and X-ray diffraction (XRD). The Raman spectra of the deposited material was obtained using a Bruker Senterra equipped with a DPSS laser (*λ* = 532 nm) at a laser power of 2 mW and with a penetration depth of <1 μm. The XRD was performed on a Bruker D8 Advance diffractometer using Cu-K*α*_1,2_ radiation (*λ* = 1.5405 Å). The lattice parameter *d*_002_ was calculated using Bragg’s equation (Equation (5)), where the *d*_002_ value relates with the wavelength (*λ*) of the X-ray and diffraction angle (*θ*) ^81^.

5 $${d}_{002}=\frac{n\lambda }{2\sin \theta }$$

The dislocation density (*δ*) of the grown ZnO nanostructures were calculated using Eq. (6), which relates with the grain size, *D*^82^. The grain size was estimated using Scherrer’s equation (Eq. 7), which corralates the grain size with the wavelength of the X-ray (*λ*), the diffraction angle *θ* and the full width at the half maximum of the XRD peaks, *β* ^83^.

6 $$\delta =\frac{1}{{D}^{2}}$$

7 $$D=\frac{k\lambda }{\beta \cos \theta }$$

The average microstrain (*ϵ*) of the ZnO nanostructures was calculated using the Stokes-Wilson equation (Eq. 8)^84^, as shown below:

8 $$\epsilon =\frac{\beta }{4\tan \theta }$$

The degree of contribution from different crystalline planes was estimated using the Harris’s analysis, by calculating the texture coefficients (TC_*hkl*_)^85^, as shown in Eq. (9).

9 $${\rm{TC}}_{hkl}=\frac{{I}_{hkl}/{I}_{hkl}^{0}}{\frac{1}{n}\mathop{\sum }\nolimits_{1}^{n}({I}_{hkl}/{I}_{hkl}^{0})}$$

Here, *I*_*hkl*_ is the measured intensity of a XRD peak and *I*${}_{hkl}^{0}$ is the corresponding peak intensity of ZnO obtained from the JCPDS card number 36-1451.

## Supplementary information

Supplemental Material
